# New evidence for the earliest ornithischian dinosaurs from Asia

**DOI:** 10.1016/j.isci.2024.111641

**Published:** 2024-12-19

**Authors:** Xi Yao, Qi Zhao, Tingcong Ren, Guangbiao Wei, Xing Xu

**Affiliations:** 1 Center for Vertebrate Evolutionary Biology, Yunnan University, Kunming 650091, China; 2 Key Laboratory of Vertebrate Evolution and Human Origins, Institute of Vertebrate Paleontology and Paleoanthropology, Beijing 100044, China; 3 Center for Excellence in Life and Paleoenvironment, Chinese Academy of Sciences, Beijing 100044, China; 4 Southeast Sichuan Geological Team, Chongqing Bureau of Geology and Minerals Exploration, Chongqing 400038, China; 5 Chongqing Institute of Paleontology, Chongqing 401122, China

**Keywords:** paleobiology, paleontology

## Abstract

The Early Jurassic ornithischian dinosaurs in Laurasia are dominated by armored dinosaurs, with other early ornithischian groups being rare. Here, a new taxon, *Archaeocursor asiaticus* gen. et sp. nov., is reported from the Lower Jurassic of southwestern China. Phylogenetic analysis places *Archaeocursor asiaticus* as the earliest-diverging ornithischian dinosaur yet discovered in Asia, albeit with weak support. Osteohistological analysis of the femoral cross-section suggests that *Archaeocursor asiaticus* was a young adult at the time of death, indicating a small body size of approximately 1 m in body length. This discovery extends the known presence of ornithischian dinosaurs in East Asia to the Pliensbachian or even late Sinemurian stages. Additionally, it points to an earlier dispersal event of Early Jurassic ornithischian dinosaurs from Gondwana to Laurasia, including East Asia, which appears to be independent of, and possibly earlier than, the dispersal of armored dinosaurs.

## Highlights

• The oldest ornithischian dinosaur from Asia is described
• It represents the earliest-diverging ornithischian dinosaur in Asia
• It suggests a potential relationship with species from Gondwana
• Histological analysis of the femur indicates that it was small in body size

## Graphical abstract

**Figure:**
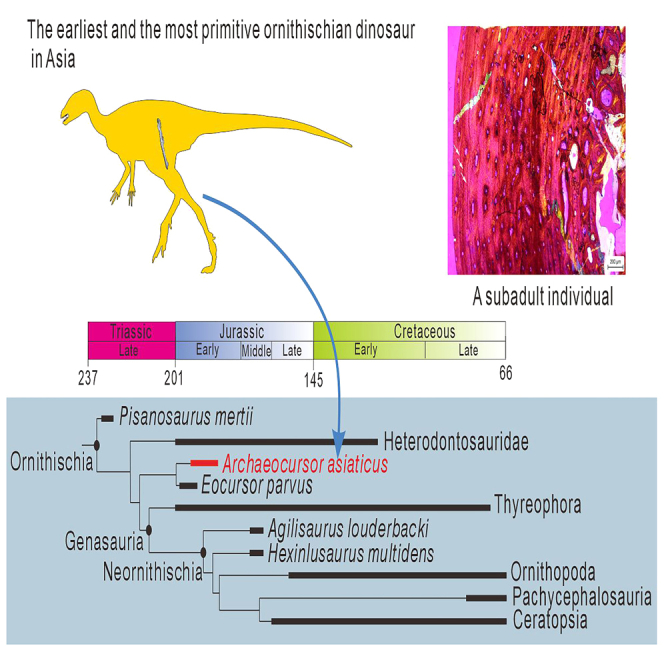

## Introduction

Ornithischia, a prominent clade of dinosaurs, diversified into various forms such as ankylosaurs, stegosaurs, hadrosaurs, ceratopsians, and pachycephalosaurs throughout the Mesozoic era.^1^^,^^2^^,^^3^ Alongside other non-avian dinosaurs, they faced extinction at the close of the Cretaceous, while their early evolutionary history remains debated. Previous reports of Triassic ornithischian dinosaurs had been considered dubious,^4^^,^^5^^,^^6^^,^^7^ thus establishing the earliest unequivocal ornithischians in the Early Jurassic. However, recent studies indicate that Silesauridae, a group of dinosauriforms from the Triassic, likely represent stem-ornithischians.^6^^,^^7^^,^^8^^,^^9^ During the Early Jurassic, ornithischian fossils are plentiful and diverse in Gondwana. Examples include *Laquintasaura venezuelae* from Venezuela,^10^ *Eocursor parvus* and *Lesothosaurus diagnosticus* from Southern Africa,^11^^,^^12^^,^^13^^,^^14^ and specialized heterodontosaurids like *Heterodontosaurus tucki*, *Abrictosaurus consors*, *Lycorhinus angustidens*, and *Pegomastax africanus* from Southern Africa and *Manidens condorensis* from Argentina.^15^^,^^16^^,^^17^^,^^18^^,^^19^^,^^20^ In contrast, the ornithischian fossil records in Laurasia during this time are less varied and primarily comprise armored dinosaurs such as *Scutellosaurus lawleri* from North America, *Yuxisaurus kopchicki* from Asia, and *Emausaurus ernsti* and *Scelidosaurus harrisonii* from Europe,^21^^,^^22^^,^^23^^,^^24^ along with fragments of thyreophorans from Germany, the USA, and China.^25^^,^^26^^,^^27^^,^^28^ An exception is an undescribed heterodontosaurid specimen from the Kayenta Formation in the USA.^15^^,^^29^ Irmis and Knoll^30^ also reported an ornithischian distal hindlimb from China’s Lower Jurassic Lufeng Formation, although its classification remains uncertain. This paper introduces *Archaeocursor asiaticus* gen. et sp. nov., a new ornithischian dinosaur based on a nearly complete left femur from the Lower Jurassic Ziliujing Formation in Chongqing Municipality, southwestern China. Notably, this taxon exhibits affinities with Gondwanan species, providing insights into the early evolution of ornithischian dinosaurs during the Early Jurassic.

## Results

### Systematic paleontology

Dinosauria Owen^31^

Ornithischia Seeley^32^

*Archaeocursor asiaticus* gen. et sp. nov.

Holotype: L01-HY999, a nearly complete left femur.

**Figure 1:**
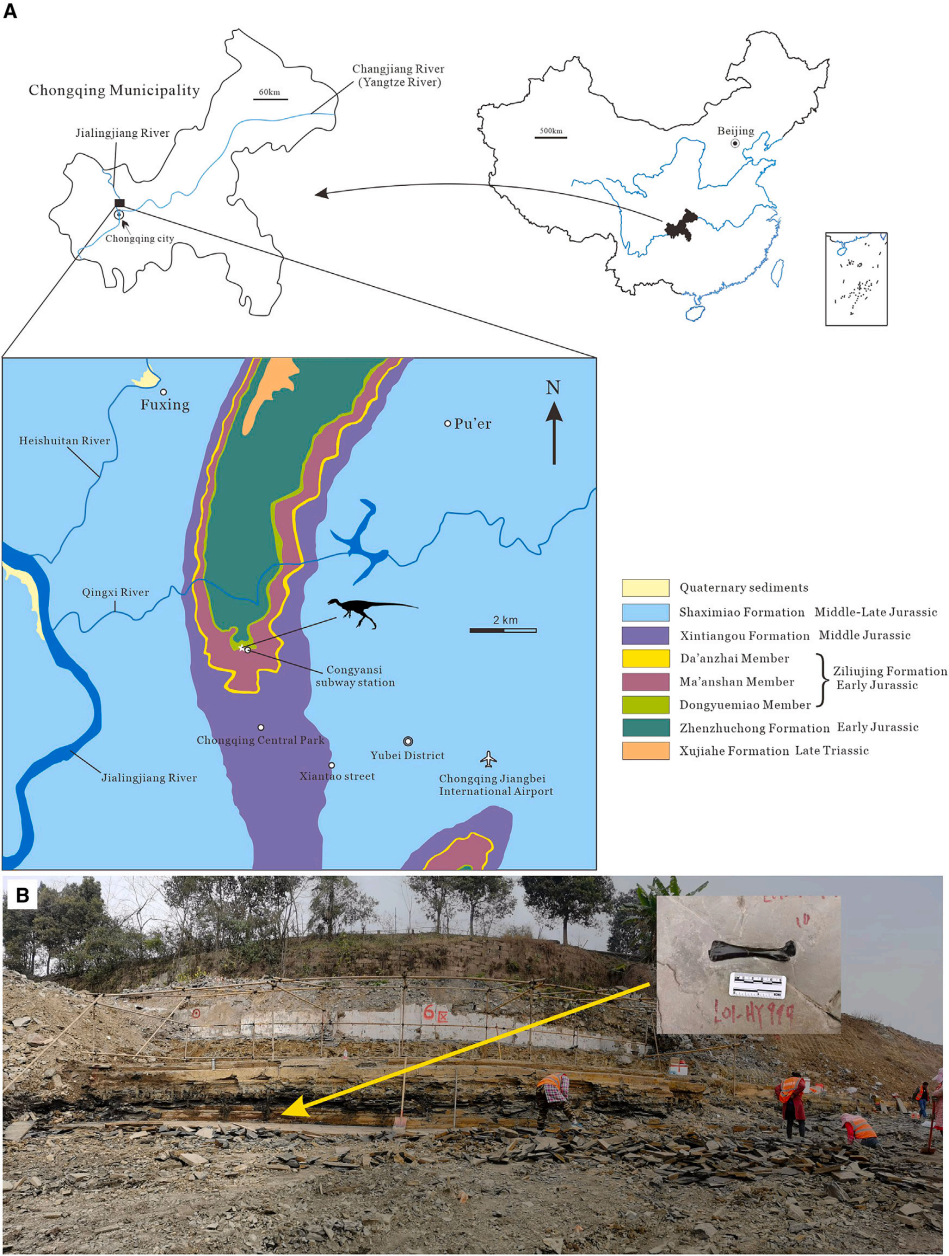
Type locality and geological setting of *Archaeocursor asiaticus* gen. et sp. nov (A) Type locality of *A*. *asiaticus* (indicated by a white star) and surrounding geological map. (B) Provenance of *A*. *asiaticus* holotype from the outcrop.

Type locality and horizon: near Congyansi subway station, GPS coordinates N 29°44'57.0" E 106°34'57.0", approximately 2 km north of Chongqing Central Park in Yubei District, Chongqing Municipality, southwestern China (Figure 1). Dongyuemiao Member of the Ziliujing Formation, Early Jurassic, late Sinemurian-Pliensbachian.^33^^,^^34^^,^^35^

Etymology: the Latin words *archaeo*, *cursor*, and *asiaticus* mean archaic, runner, and Asian, respectively. Together, the binomial name means an old runner from Asia.

Diagnosis: a small-bodied (∼1 m in body length) ornithischian dinosaur distinguished by the following features, with autapomorphies marked by asterisks (∗): ∗femoral proximal surface marked by an anterolateral-posteromedially oriented ridge; ∗the greater trochanter expands anteroposteriorly to approximately the same width as the femoral head but remains shorter than the anterior trochanter; ∗the anterior surface of the medial condyle is sculpted, featuring an associated depression on its medial surface; ∗lateral condyle occupying roughly 2/3 of the distal transverse width; lateral condyle distinctly inset medially (also seen in *Laquintasaura venezuelae* and *Yuxisaurus kopchicki*); anterior intercondylar groove visible in distal view (also seen in *Scelidosaurus harrisonii*).

**Figure 2:**
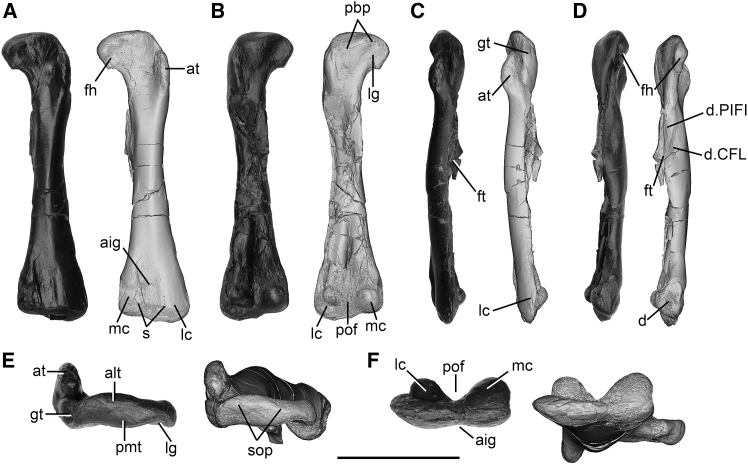
L01-HY999, the holotype left femur of *Archaeocursor asiaticus* (photograph and CT image) (A) Anterior view. (B) Posterior view. (C) Lateral view. (D) Medial view. (E) Proximal view. (F) Distal view. aig, anterior intercondylar groove; alt, anterolateral tuber; at, anterior trochanter; d, depression; d. CFL, depression for the attachment of M. caudofemoralis longus; d. PIFI, depression for the attachment of M. pubo-ischio-femoralis internus; fh, femoral head; ft, fourth trochanter; gt, greater trochanter; lc, lateral condyle; lg, ligament groove; mc, medial condyle; pbp, posterior boundary of the proximal end; pmt, posteromedial tuber; pof, popliteal fossa; s, suture; sop, summit of the proximal end. Scale bar: 30 mm.

Description and comparison: this specimen is nearly complete with a total length of 93 mm, though its posterior surface has been somewhat crushed. In anterior view, the femur appears straight, but in lateral view, it exhibits a slight anterior bowing typical of early ornithischian dinosaurs (Figures 2A and 2C). The proximal view reveals that the femoral head is anteromedially inclined at approximately 20° relative to the transverse axis of the distal condyles, a feature shared with early ornithischian *Eocursor parvus*, contrasting with species like *Lesothosaurus diagnosticus* (NHMUK PV RU B17) where both are in the same plane.^36^^,^^37^ The femoral head projects ventromedially with a straight medial margin and a smoothly convex dorsal margin (Figure 2A). On the posterior surface, lateral to the femoral head, there is a broad sulcus for attaching the ligament *femoral capitis*, forming a broad concavity in proximal view (Figures 2B and 2E). Opposite this sulcus on the anterior surface, there is a shallow depression. The proximal surface features well-developed tubers: the posteromedial tuber is broad and mound-like, positioned centrally along the posterior margin and separated from the greater trochanter by a depressed, posteroventrally sloped surface, which corresponds to the *facies articularis antitrochanterica* seen in other dinosauriforms.^38^ The anterolateral tuber on the anterior surface is broadly convex, with its central portion lateral to the posteromedial tuber and merging with the greater trochanter laterally. This configuration creates an anteroposteriorly constricted region between the femoral head and the main surface in proximal view (Figure 2E). In contrast to other early dinosaurs where the femoral proximal surface is excavated by a transverse groove,^12^^,^^29^^,^^36^^,^^39^ in *Archaeocursor asiaticus*, the summit of the femoral proximal end is represented by a curved ridge curving from the anterolateral corner to the posteromedial corner, dividing the proximal surface into anterior and posterior regions (Figure 2E). This ridge differs from the proximal groove seen in other dinosaurs and suggests a unique morphology in *Archaeocursor asiaticus*. In comparison with early ornithischian dinosaurs, the femoral proximal end of *Archaeocursor asiaticus* exhibits pronounced anteroposterior compression.

The greater trochanter extends anteroposteriorly to subequal the femoral head in length along its lateral margin, akin to *Scelidosaurus harrisonii* and *Sanxiasaurus modaoxiensis*.^39^^,^^40^ This contrasts with the condition in *Lesothosaurus diagnosticus*, *Eocursor parvus*, and *Scutellosaurus lawleri*, where the greater trochanter is shorter relative to the femoral head.^10^^,^^27^^,^^41^ In other late-diverging ornithischians, such as *Hexinlusaurus multidens*, *Nanosaurus agilis*, and *Minimocursor phunoiensis*, the greater trochanter greatly expands anteroposteriorly to exceed that of the femoral head.^42^^,^^43^^,^^44^ The trochanter expands laterally to create a continuous convex external surface, predominantly along the lateral margin, with some extension onto the posterior surface (Figures 2B, 2C, and 2E).

The anterior trochanter is finger-like in anterior view, located on the lateral margin of the femoral shaft, transversely thickened, and sub-elliptical in outline (Figure 2A). Its dorsal end is rounded and distally positioned from the greater trochanter but is still higher than the ventral extremity of the femoral head (Figure 2A). This feature is widespread among early ornithischians; however, in others such as *Nanosaurus agilis*, *Jeholosaurus shangyuanensis*, and heterodontosaurids *Heterodontosaurus tucki*, *Abrictosaurus consors*, and *Fruitadens haagarorum*, the anterior trochanter reaches the level of the greater trochanter.^15^^,^^44^^,^^45^^,^^46^^,^^47^ In anterior view, the anterior trochanter is in line with the greater trochanter, whereas in *Lesothosaurus diagnosticus*, *Laquintasaura venezuelae,* and *Scutellosaurus lawleri*, the greater trochanter inclines medially from the anterior trochanter in an angle.^10^^,^^29^^,^^37^^,^^41^ In lateral view, the anterior trochanter is broad and wing-like with a convex anterior margin. Posteriorly, it is close to the greater trochanter but is still separated from the latter by a groove, which could be seen in proximal view as in early ornithischian dinosaur *Eocursor parvus*,^36^ in contrast to the late-diverging ornithischian *Jeholosaurus shangyuanensis* in which they are fused (Figure 2C).^45^ Additionally, in *Archaeocursor asiaticus*, the anterior trochanter extends anteroposteriorly more than the greater trochanter, similar to *Eocursor parvus* and *Lesothosaurus diagnosticus*.^12^^,^^36^ In contrast, in all other ornithischians examined, such as *Hexinlusaurus multidens* and *Minimocursor phunoiensis*, the greater trochanter extends significantly further in anteroposterior length compared to the anterior trochanter. Notably, in *Sanxiasaurus modaoxiensis*, their lengths are nearly equal.^39^^,^^42^^,^^43^ The ventral margin of the anterior trochanter transitions smoothly into the main shaft, and the anterior surface of the femoral shaft is smoothly convex (Figure 2A). In anterior view, it is waisted at mid-length, contributing to its slender morphology, similar to *Eocursor parvus*.^36^ The fourth trochanter is incompletely preserved but shows a well-developed flange. The distal-most portion is displaced; however, based on the remaining part, the original morphology suggests it was pendent. It arises from the posteromedial margin of the shaft, aligning with the ventral extremity of the anterior trochanter, and extends downward for approximately 25 mm, slightly surpassing the mid-length of the shaft. In dorsal view, it primarily extends posteriorly but slightly medially, visible also in anterior view (Figures 2A–2D). In medial view, the dorsal margin of the fourth trochanter exhibits a concave and dorsally curved shape. On the medial surface, centrally positioned on the fourth trochanter, there is an oval, rough depression that extends anterodorsally to posteroventrally. Situated posterodorsally to this depression is another elongated, rough area, parallel to the former but separated by a gentle ridge. This ridge extends anterodorsally and merges with the medial margin of the femoral shaft (Figure 2D). This ridge configuration resembles that observed in hadrosaurid *Orthomerus dolloi*, as well as hadrosaurs *Edmontosaurus regalis* and *Hypacrosaurus altispinus*, albeit directed anteroventrally in those species.^48^^,^^49^^,^^50^ These two rough areas likely indicate the attachment sites for the muscles caudofemoralis longus (CFL) and pubo-ischio-femoralis internus (PIFI), as observed in *Lesothosaurus diagnosticus*.^49^ In posterior view, the distal portion of the fourth trochanter curves slightly laterally.

The distal end of the femur shows a marked transverse expansion relative to the shaft. The middle portion of the distal anterior surface forms a distinct broad intercondylar groove in anterior view, which is also visible in distal view, whereas the surfaces medial and lateral to this groove are both convex. Fine longitudinal striations mark the intercondylar groove, whereas the surface medial to it is irregularly sculpted and has a “stepped” margin from this groove (Figure 2A). The sculpted medial surface is unique to this species since this surface is usually smooth in other ornithischians. The lateral condyle is more distally projected than the medial condyle, resulting in an oblique, medially inclined, slightly concave distal margin of the femur (Figure 2A), a characteristic shared with other early ornithischians.^51^ The lateral condyle is prominent in anterior view, occupying approximately two-thirds of the transverse width, whereas in other ornithischians like *Eocursor parvus*, *Lesothosaurus diagnosticus*, *Scelidosaurus harrisonii*, and *Hexinlusaurus multidens*, the lateral condyle is generally equal to the medial condyle in width.^36^^,^^37^^,^^40^^,^^43^

In posterior view, the lateral condyle is oblique and ovoid, markedly inset medially and dorsally from the lateral and distal margins (Figure 2B), a feature also seen prominently in *Laquintasaura venezuelae* among ornithischians.^10^ Above the lateral condyle, a broad ridge continues dorsally. The medial condyle is nearly bulbous in posterior view, significantly larger than the lateral condyle, and exhibits a rounded or elliptical shape (Figure 2B). The popliteal fossa is elongated, extending to more than one-quarter of the total femoral length, a condition observed in several silesauridae.^52^ The fossa is depressed and becomes deeper dorsally in posterior view, with the condyles widely separated from each other.

The distal surface of the femur is wider transversely than anteroposteriorly long, subdivided into anterior and posterior parts by a transverse ridge (Figure 2A). In distal view, both posterior protuberances are broad, with the lateral condyle extending posterolaterally and the medial condyle posteromedially, a feature also observed in *Eocursor parvus*.^36^ The condyles extend approximately equally posteriorly in distal view as in other early ornithischians. In distal view, the popliteal fossa is broad and deep, and there is a shallow intercondylar groove on the anterior surface (Figure 2F). This groove appears to vary in development among early ornithischian dinosaurs, such as *Eocursor parvus*, *Lesothosaurus diagnosticus*, *Scutellosaurus lawleri*, and *Scelidosaurus harrisonii*, but is only exposed distally in *Scelidosaurus harrisonii*.^29^^,^^36^^,^^37^^,^^40^^,^^45^ The medial surface of the medial condyle exhibits a sub-elliptical rough depression, distinctly marked from the surrounding areas (Figure 2D).

**Figure 3:**
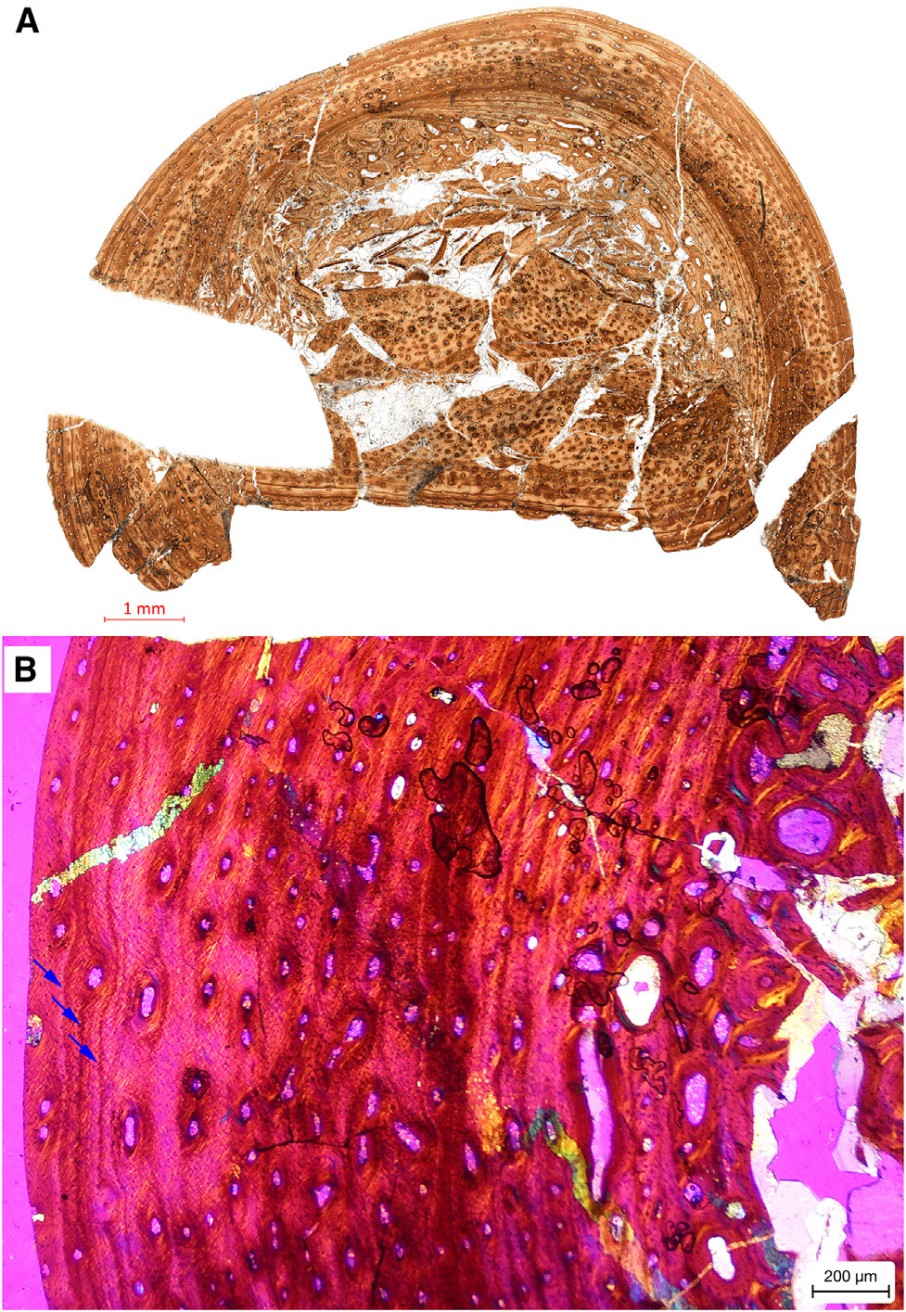
Osteohistological section of the left femur of *Archaeocursor asiaticus* holotype (A) Complete section under normal polarized light. (B) Close-up of cortex under the cross-polarized light with lambda filter. The arrows indicate the tightly packed LAGs.

Osteohistology: the transverse thin section reveals a compact bone wall thickness ranging between 1.6 and 1.8 mm. The primary cortex exhibits characteristics of parallel-fibred bone with longitudinal vascular canals (Figures 3A and 3B). Vascular canal density is notably high in the inner and middle cortex, gradually diminishing toward the outer cortex (Figures 3A and 3B). Secondary osteons are prominently distributed near the periosteal-endosteal interface of the compacted coarse cancellous bone (CCCB), with a prevalence of large secondary osteons displaying more than three layers of osteocyte lacunae surrounding the vascular canals. Some secondary osteons exhibit signs of subsequent remodeling by successive generations (Figure 3A). Conversely, the middle and outer cortex predominantly contains smaller primary osteons characterized by a single layer of osteocyte lacunae around the vascular canals. Distinct lines of arrested growth (LAGs), resembling an external fundamental system (EFS), are densely packed near the outer bone surface. Primary osteons are positioned exterior to these densely packed LAGs. The outermost matrix demonstrates flattened osteocyte lacunae, indicative of markedly reduced growth rates during this phase (Figure 3B). Based on these histological features, the individual is classified as an early adult at the time of death, yet had not attained somatic maturity. This interpretation is supported by the presence of LAGs and the observed deceleration in growth rate within the outer bone layers.

**Figure 4:**
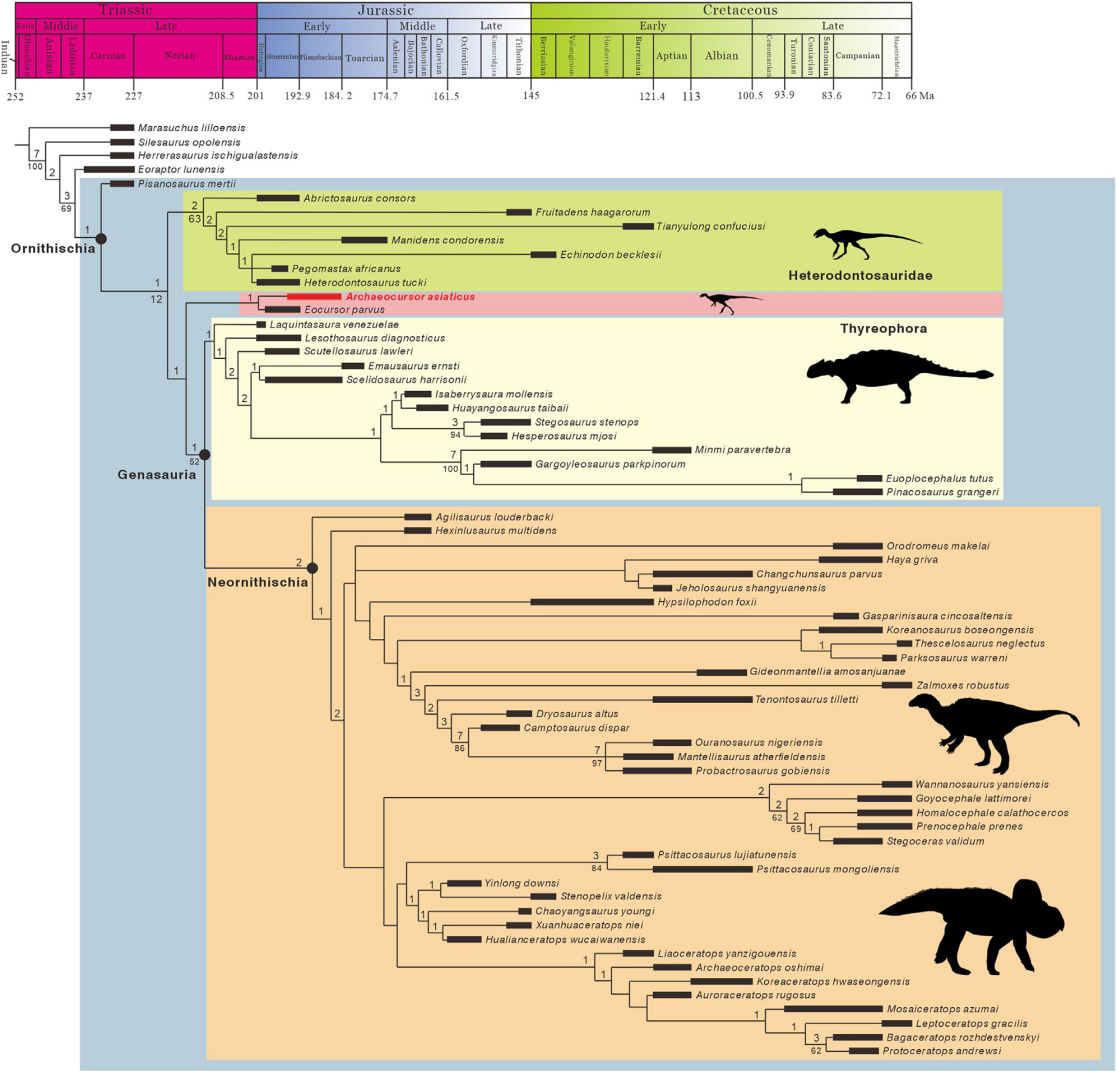
Reduced consensus tree of 6,672 most parsimonious trees illustrating the phylogenetic position of *Archaeocursor asiaticus* gen. et sp. nov. within ornithischian dinosaurs Seven unstable taxa, *Yandusaurus hongheensis*, *Yueosaurus tiantaiensis*, *Zephyrosaurus schaffi*, *Aquilops americanus*, *Yamaceratops dorngobiensis*, *Albalophosaurus yamaguchiorum*, and *Micropachycephalosaurus hongtuyanensis*, are excluded *a posterior*. Values beneath nodes represent bootstrap proportions, and those above nodes indicate Bremer support.

Phylogenetic analysis: the result produced 6,672 most parsimonious trees with a tree length of 1,226 steps, CI (consistency index) 0.71, and RI (retention index) 0.36. In agreement with previous result,^53^ *Pisanosaurus mertii* was recovered as the earliest-diverging ornithischian dinosaur, and Heterodontosauridae as a well-established earliest-diverging group. Thyreophora forms sister group to Neornithischia in the topology, and together they form a clade Genasauria. The most surprising aspect is that, although weakly supported, *Archaeocursor asiaticus* forms a monophyletic group with *Eocursor parvus*, lying immediately outside Genasauria. This clade is supported by a single unambiguous synapomorphy (character 382, 1) that the anterior trochanter is closely appressed but unfused with the greater trochanter, where a dorsal view reveals a slight space, but is invisible in lateral view. The usually volatile early ornithischian dinosaurs *Lesothosaurus diagnosticus* and *Laquintasaura venezuelae* were recovered successively as early-diverging thyreophoran dinosaurs. To better resolve the relationships among neornithischian dinosaurs, seven “wildcard taxa” (*Yandusaurus hongheensis*, *Yueosaurus tiantaiensis*, *Zephyrosaurus schaffi*, *Aquilops americanus*, *Yamaceratops dorngobiensis*, *Albalophosaurus yamaguchiorum*, *Micropachycephalosaurus hongtuyanensis*) were identified using the TNT script *IterPCR* and were excluded in the final consensus. The reduced consensus provides a better-resolved topology (Figure 4).

## Discussion

*Archaeocursor asiaticus* is identified as an ornithischian dinosaur based on distinctive morphological features, namely a broad, wing-like anterior trochanter and a well-developed flange-like fourth trochanter, which differentiate it within Ornithischia.^1^^,^^2^ Osteohistological analysis of the holotype suggests it belonged to a young adult, characterized by its small femoral length, indicative of a diminutive body size.

Ornithischian fossils are scarce in Early Jurassic deposits of Asia, primarily reported from the contemporaneous Lower Jurassic Lufeng and Fengjiahe formations in Yunnan province, also in southwestern China. Notably, *Yuxisaurus kopchicki*, an early armored dinosaur from the Fengjiahe Formation, is the sole formally named Early Jurassic ornithischian from Asia.^24^ The femur of the holotype is poorly preserved, and only the distal portion remains. *Yuxisaurus kopchicki* has a femoral autapomorphy: a marked depression on the medial surface, which forms a distinct concavity in distal view. However, this feature is absent in *Archaeocursor asiaticus*, and the medial surface of the distal femur is only weakly depressed in medial and distal views (Figures 2D and 2F). Several distinct anatomical differences can be observed between these two taxa. Firstly, *Archaeocursor asiaticus* exhibits a robust ridge immediately above the lateral condyle in posterior view, a feature absent in *Yuxisaurus kopchicki*. Additionally, the femoral lateral condyle in *Archaeocursor asiaticus* protrudes more distally compared to the medial condyle, resulting in an oblique distal margin; in contrast, both condyles are equally extensive in *Yuxisaurus kopchicki*. Furthermore, the orientation of the lateral condyle differs between the two taxa: it is posterolaterally oriented in distal view in *Archaeocursor asiaticus*, whereas it is posteromedially directed in *Yuxisaurus kopchicki*. Finally, the popliteal fossa of *Archaeocursor asiaticus* appears nearly “V” shaped in distal view, whereas in *Yuxisaurus kopchicki*, it is deep and narrow, forming a “U” shape.

Other reported ornithischian specimens from the Lower Jurassic of Asia originate exclusively from the Lufeng Formation. IVPP V15311 comprises a partial right dentary with associated cranial fragments. Initially designated as an ankylosaurian named “*Bienosaurus lufengensis*,” it is now considered an indeterminate thyreophoran dinosaur.^27^ Similarly, FMNH CUP 2088 includes a partial left dentary with fragments of the quadrate and surangular-articular. Initially identified as a “hypsilophodontid” named “*Tatisaurus oehleri*,” this specimen also now falls under the category of indeterminate thyreophoran dinosaur^28^; Irmis and Knoll^30^ reported FMNH CUP 2338, a partial hindlimb from the Lufeng Formation attributed to an indeterminate ornithischian dinosaur. Unfortunately, none of these specimens include preserved portions of the femur, thus preventing direct comparison. In terms of size, *Archaeocursor asiaticus* appears to be similar in size to *Eocursor parvus*, an early-diverging ornithischian dinosaur from the Lower Jurassic of South Africa, based on their comparable femoral length (93 mm for *Archaeocursor asiaticus* versus 107 mm for *Eocursor parvus*). The dentary of *Eocursor parvus* measures approximately 4.8 cm in anteroposterior length, and the distal end of the tibia is around 3.06 cm in transverse width.^36^ These dimensions are slightly smaller than those of “*Bienosaurus lufengensis*” (dentary approximately 5.96 cm) and “*Tatisaurus oehleri*” (dentary approximately 5.05 cm) but somewhat larger than FMNH CUP 2338 (tibial distal width approximately 2.63 cm). In comparison, *Yuxisaurus kopchicki* has a femoral distal transverse width of approximately 18.10 cm, whereas this dimension is only 2.92 cm in *Archaeocursor asiaticus*. In summary, *Archaeocursor asiaticus* is markedly smaller than *Yuxisaurus kopchicki* from the Fengjiahe Formation but aligns in size to the three ornithischian specimens from the Lufeng Formation.

The discovery of *Archaeocursor asiaticus* in the Ziliujing Formation extends the known range of small-bodied ornithischians (body length ∼1 m) in East Asia to the Early Jurassic. Ornithischian dinosaurs originated from Gondwana and migrated to the Northern continent in the Early Jurassic,^24^ but the timing of their arrival to East Asia is obscure. The stratigraphical age of *Yuxisaurus kopchicki* is poorly constrained, with magnetostratigraphic evidence from the lateral equivalent Lufeng Formation suggesting a range from late Sinemurian to Toarcian stages.^24^ On the other hand, *Archaeocursor asiaticus* is found in the Dongyuemiao Member of the Ziliujing Formation, which is generally accepted to be of Pliensbachian age based on regional stratigraphy and geological context.^34^^,^^35^ Geochemical and palynological data even suggest a late Sinemurian to early Pliensbachian age for this member.^33^ The uppermost layer of the Ziliujing Formation, the Da’anzhai Member, has been solidly dated to the Toarcian stage through various lines of evidence such as palynostratigraphy, Re-Os chronology, and chemostratigraphy.^54^ Therefore, the assignment of a Pliensbachian age to the Dongyuemiao Member seems plausible. If this age assignment is correct, the discovery of *Archaeocursor asiaticus* indicates that ornithischian dinosaurs arrived in East Asia during the Pliensbachian or even late Sinemurian, supporting the hypothesis of a rapid spread of ornithischian dinosaurs during the Early Jurassic,^24^ and provides further refinement of this time interval.

The phylogenetic analysis highlights *Archaeocursor asiaticus* as the earliest-diverging ornithischian dinosaur discovered in Asia, marking a significant contribution to understanding their biogeographical distribution. Heterodontosaurid dinosaurs, recognized as the earliest ornithischian group, are well documented in the Early Jurassic of Gondwana, including South Africa and Argentina. The presence of undescribed heterodontosaurid remains in the Kayenta Formation of the USA suggests their global distribution across continents during this period,^15^ indicating at least one dispersal event of ornithischian dinosaurs between Gondwana and Laurasia. The origin of thyreophoran dinosaurs remains debated. If taxa like *Lesothosaurus diagnosticus* or *Laquintasaura venezuelae* are identified as early-diverging thyreophorans as in the current analysis and also some previous studies,^1^^,^^2^^,^^37^^,^^55^^,^^56^ this group should have dispersed at least once from Gondwana to Laurasia in the Early Jurassic and is also supposed to disperse from North America to Asia subsequently, given the geographic position of East Asia during this period^57^ and the clear presence of armored dinosaurs in this region at this time.^24^^,^^27^^,^^28^ Even if, alternatively, *Lesothosaurus diagnosticus* and *Laquintasaura venezuelae* were not thyreophoran dinosaurs as recovered by some other studies,^7^^,^^10^^,^^11^^,^^58^^,^^59^^,^^60^ the primitive nature of *Scutellosaurus lawleri* suggests thyreophorans also dispersed from North America to East Asia during the Early Jurassic.

The new phylogenetic topology proposed in this study suggests an additional independent dispersal of ornithischian dinosaurs into East Asia during the Early Jurassic. The close relationship between *Archaeocursor asiaticus* and *Eocursor parvus*, despite their distant habitats, suggests a probable origin from Gondwana, followed by northward migration to Laurasia and eventually to East Asia during the Pliensbachian. This timing might precede the arrival of armored dinosaurs in the region. Moreover, this new topology hypothesizes the existence of a previously unrecognized cosmopolitan clade of early ornithischian dinosaurs, positioned phylogenetically between Heterodontosauridae and Thyreophora. Nevertheless, due to the fragmentary nature of the *Archaeocursor asiaticus* holotype, support for this clade remains tentative, awaiting further fossil discoveries.

### Limitations of the study

This research relies on a single fossil femur that can be confidently identified as belonging to an early diverging ornithischian dinosaur; nevertheless, its evolutionary connections with other early diverging ornithischians remain inadequately understood. The two distinct data matrices utilized in this research produced comparable results, albeit with slight discrepancies, especially concerning its sister taxon relationship with *Eocursor parvus*, which is only weakly substantiated. The discovery of additional, more complete specimens in the future will be essential in clarifying these relationships.

## Resource availability

### Lead contact

Requests for further information and resources should be directed to and will be fulfilled by the lead contact, Xi Yao (yaoxi@ynu.edu.cn).

### Materials availability

• The described specimen is housed in Chongqing Municipal Bureau of Planning and Natural Resources (Chongqing, China). Access to the fossil specimen will be made available on request for qualified researchers.
• This study did not generate new unique reagents.

### Data and code availability

• The data matrix used in the phylogenetic analysis is available in https://doi.org/10.5061/dryad.08kprr5d0.
• This paper does not report original code.

## Acknowledgments

We express our gratitude to Qingdong Wang and Qiang Hu for their discovery and expert preparation of this delicate specimen. Dr. Huijuan Mai provided assistance with the CT scanning process. Dr. Marcos Becerra and an anonymous reviewer provided constructive comments that greatly improved the manuscript. This research was generously supported by the Yunnan Revitalization Talent Support Program (202305AB350006) and the 10.13039/501100001809National Natural Science Foundation of China (grant No. 42288201).

## Author contributions

X.X. and G.W. designed research; X.Y., Q.Z., and T.R. performed research; X.Y., Q.Z., and T.R. analyzed data; X.Y., Q.Z., X.X., T.R., and G.W. wrote the paper. All authors approved the manuscript for publication.

## Declaration of interests

The authors declare no competing interests.

## Declaration of generative AI and AI-assisted technologies in the writing process

During the preparation of this work the authors used ChatGPT in order to improve language and readability. After using this tool/service, the authors reviewed and edited the content as needed and take full responsibility for the content of the publication.

## STAR★Methods

### Key resources table

**Table:** 

REAGENT or RESOURCE	SOURCE	IDENTIFIER
**Deposited data**		

Data matrix for phylogenetic analysis	This paper	https://doi.org/10.5061/dryad.08kprr5d0
Supplemental information	This paper	https://doi.org/10.5281/zenodo.14491595

**Software and algorithms**		

TNT version 1.1	Goloboff et al.^61^	http://www.lillo.org.ar/phylogeny/tnt/

### Method details

#### CT scanning

The specimen underwent CT scanning using a Micro-X ray-CT system, specifically the Xradia 520 Versa from Carl Zeiss X-ray Microscopy, Inc., located in Pleasanton, USA. The scanning procedure was conducted at the Yunnan Key Laboratory for Palaeobiology, Institute of Palaeontology, Yunnan University, Kunming, China. Scanning parameters are as follows: Beam strength was 80kV/7w with no filter; the exposure time of each projection was 0.45 seconds; the pixel size was 30.88um. A total of 3393 radiographic images (projections) were acquired during the scan, and these images were saved as TIFF stacks. Subsequently, the image data were processed and reconstructed in Mimics software (Version 10.01) to generate a three-dimensional representation of the specimen.

#### Histological analysis

A bone sample was taken near the mid-diaphysis of the femur of *Archaeocursor asiaticus*. The sample was embedded in one-component resin (EXAKT Technovit 7200), which was then hardened in a light polymerization device (EXAKT 520). A thin cross-section was cut to a thickness of about 200 μm using a high-precision circular saw (EXAKT 300CP). It was ground down to the final thickness of 50–80 μm using the EXAKT 400CS grinding system until the desired optical transparency was obtained. The histological section was examined under a polarized light microscope (ZEISS Primotech) and photographed with an integrated 3megapixel Camera for ZEISS Primotech using the Labscope Material App for iPad OS.

#### Institutional abbreviations

**FMNH CUP**, Field Museum of Natural History (Catholic University of Peking collection), Chicago, USA; **IVPP**, Institute of Vertebrate Paleontology and Paleoanthropology, Chinese Academy of Sciences, Beijing, China; **NHMUK**, Natural History Museum, London, UK.

#### Nomenclatural acts

The nomenclatural acts performed in this work have been registered in ZooBank. The Life Science Identifiers for this publication are: urn:lsid:zoobank.org:act:0B0AF693-B615-46DD-AE67-F5E9DD53311F.

#### Geological setting and locality information

The new specimen was recovered from Xiantao Street in Yubei District, located 21 km north of Chongqing City, during a paleontological salvage operation conducted by the Southeast Sichuan Geological Team, Chongqing Bureau of Geology and Minerals Exploration, in 2022. The fossils were unearthed during construction of a residential community after the overlying Ma’anshan Member rocks were removed. The exposed fossil-bearing outcrop consists of greyish-black, thin to medium-layered calcareous marlstones, reaching a total depth of approximately 3 meters. Alongside the newly discovered dinosaur, the site yielded several well-preserved fossils including plesiosaur vertebrae and ribs, fish fossils, coprolites, plant remains, and ostracods. Geologically, this unit belongs to the Dongyuemiao Member of the Lower Jurassic Ziliujing Formation (Figure 1). The Ziliujing Formation is widely distributed in the Sichuan Basin and is positioned between the underlying Lower Jurassic Zhenzhuchong Formation and the overlying Middle Jurassic Xintiangou Formation. It comprises three continuous members, arranged in ascending order: Dongyuemiao Member, Ma’anshan Member, and Da’anzhai Member.^62^ While the Ziliujing Formation is less fossil-rich than the contemporaneous Lufeng Formation in Yunnan Province, it has yielded significant discoveries. These include early-diverging sauropodomorph dinosaur cf. *Lufengosaurus magnus* (Da’anzhai Member), sauropod dinosaurs like *Gongxianosaurus shibeiensis* (Dongyuemiao Member), and *Sanpasaurus yaoi* (Ma’anshan Member), as well as various dinosaur footprints.^63^^,^^64^^,^^65^^,^^66^^,^^67^^,^^68^^,^^69^^,^^70^^,^^71^ A sauropod dinosaur referred to as “*Yibinosaurus zoui*” was named based on an incomplete skeleton from the Dongyuemiao Member in Shibei Township, Gongxian County, although it remains inadequately described and is considered a *nomen nudum* today.^72^ Additionally, fragmentary dinosaur remains from the Ziliujing Formation have been tentatively attributed to different taxa. For instance, Young^65^ assigned four isolated spines from Changshanling in Weiyuan County (Ma’anshan Member) to Stegosauria indet., and identified four vertebrae and a metatarsal fragment from a nearby locality (also Ma’anshan Member) as Coelurosauria indet.; Dong et al.^73^ reported vertebral and limb elements belonging to Cetiosaurinae from Huangshiban in Weiyuan county (Ma’anshan Member), while Luo and Wang^67^ mentioned theropod vertebrae and teeth from the locality where *Gongxianosaurus* fossils were found; Peng et al.^74^ referred isolated vertebrae and limb bones from Zigong City (Da’anzhai Member) to Plateosauridae and Cetiosauridae, respectively.

### Quantification and statistical analysis

#### Phylogenetic analysis

To assess the phylogenetic position of *Archaeocursor asiaticus* within ornithischian dinosaurs, a phylogenetic analysis was conducted using the data matrix from Han et al*.*^53^ Characters 353 and 354 from the original character list have been divided into four distinct characters (353, 354, 381, and 382) in the updated character lists. *Archaeocursor asiaticus* could be scored for only eleven characters, accounting for three percent of the total characters (see supplemental information). This resulted in a final dataset with 382 characters from 73 operational taxonomic units. Following the original methodology, twenty-one characters were ordered (2, 23, 31, 39, 125, 163, 196, 203, 204, 222, 227, 238, 243, 247, 268, 292, 296, 302, 306, 320, 361), and the reformulated character 353 was also ordered. The dataset was analyzed using TNT 1.1 with equally weighted characters and traditional search methods, employing the tree bisection-reconnection (TBR) swapping algorithm.^61^ The search involved 1000 replicates, with a maximum of 100 trees saved per replicate, and a second round of TBR was conducted to find all the most parsimonious trees. Standard bootstrap and Bremer support were calculated to assess the robustness of each node. *Archaeocursor asiaticus* was also scored at the Fonseca et al.^9^ dataset, with similar results presented at the supplemental information.

### Additional resources

Supplemental information: https://doi.org/10.5281/zenodo.14491595.
